# Cerebro-Spinal Meningitis

**Published:** 1849-07

**Authors:** H. Roberts

**Affiliations:** Elbridge


ART. IV. — *Cerebro-spinal meningitis.* Communicated in a letter to Prof. LEE, by H. ROBERTS, M. D.

ELBRIDGE, May 14th, 1849.

DEAR SIR :—

By request of my son, I take the liberty to write to you on the subject of a malignant epidemic which is prevailing in our county. Pressing professional engagements prevent my giving a minute report of cases under, my care. I will give a brief synopsis of one case which has continued longer than any other under my treatment.

William Hunt, aged 11 years, of good general health, went to bed in his usual good health, April 9th, and was discovered to be in convulsions at 2 A. M. Being in attendance on a patient in labor, I did not see him until 4 A. M. Found him still in violent convulsions, with raving delirium in the interval of spasms; pulse quick, but weak ; skin dry and hot ; countenance pallid, and of a death-like appearance. Administered Ipecac 10 gr., yellow oxyde of mercury 1 gr., which operated in 5 minutes as an emetic. After one hour, the symptoms remaining unchanged, I gave another dose of the emetic, which operated as emetic and cathartic. In an hour the spasms ceased, and the delirium relaxed into a moaning stupor. Through the day I applied a blister to the nape of the neck, mustard poultices to the feet, alternated with the warm foot bath ; frictions the whole length of the spine, with liniment of olive oil and aqua Ammoniæ *a a* 2 parts ; spts. of turpentine 1 part ; gave full cathartic dose of calomel, which operated well, producing dejections of dark tar-like bile ; infused hops in vinegar and water, fomented the head and whole body frequently with this infusion. Through the afternoon and evening there was a general improvement in all the symptoms. At 12, the 2nd night, excitement and spasms again returned with wild delirium. Being again absent with the sick I did not see him until after 1 o'clock A. M. I found him the same as on the night previous; gave camphor and Dover's powder, and anodyne diaphoretic drinks. The spasms soon subsided.

For the following 10 days the patient remained insensible, or nearly so, being unable to move the limbs or turn himself in bed ; continued to give one full dose of calomel each day, applied one blister every second day either to the spine or back of the head ; continued the fomentations and liniment, with Dover's powder and diaphoretic drinks ; kept up a gentle perspiration by this course most of the time. On the 11th day every symptom of diseased action was gone, and so remained three days. The

15th day a gradual loss of consciousness and sensibility, and of the ability to move the legs or turn in bed. Gave grs. v of calomel every 4 hours, combined with diaphoretics ; resumed the use of fomentations, frictions, the bath, &c.; on the 20th day a blister covering the back of the head ; as this vesicated, every symptom of disease again disappeared. The mercury has not produced its specific effect on the glands, that is, no sore mouth. On the 24th day a gradual return of the disease as before. Applied an antimonial emplastrum to the spine, which, in two days produced fine antimonial sores the whole length of the spine. Used the electro magnetism daily, applying one button to the foot, passing the other along the spine. No increased pulse or fever this time ; the patient gradually wasting away. Ordered a nourishing diet, and gave brandy and quinine, which produces no fever or unpleasant symptoms. For some days past have given no medicine, continuing the electro magnetism and a nutritive diet. The lad now lies as in a quiet sleep all of the time except when aroused to take food or drink, his flesh nearly all gone. Under these circumstances will you have the goodness to suggest a mode of treatment which will probably restore my patient?

I would say in relation to the epidemic now prevailing, that the inflammation does not always commence in the brain. One of my patients was taken with severe pain in the arm, then the leg, then the side, and lastly in the head, each place of pain successively mortifying ; this patient lived 48 hours.

I am now attending one whose fingers are highly inflamed from the end to the root of the nails, and one who has one spot of inflammation on the side.

Yours truly,

H. ROBERTS.

